# Impact of arm position and load on upper and lower esophageal sphincter pressures

**DOI:** 10.3389/fphys.2026.1735342

**Published:** 2026-03-19

**Authors:** Petr Bitnar, Adam Kurka, Andrew Busch, Tereza Stehnova, Katerina Madle, Jan Stovicek, Alena Kobesova

**Affiliations:** 1 Department of Rehabilitation and Sports Medicine, Second Faculty of Medicine, Charles University and Motol University Hospital, Prague, Czechia; 2 Department of Health and Human Kinetics, Ohio Wesleyan University, Delaware, OH, United States; 3 Department of Internal Medicine, Second Faculty of Medicine, Charles University and Motol University Hospital, Prague, Czechia

**Keywords:** diaphragm, lower esophageal sphincter, manometry, posture, upper esophageal sphincter

## Abstract

**Background:**

The diaphragm contributes to respiration, postural stabilization, and regulation of esophageal sphincter pressures through its crural portion. Although its dual respiratory–postural role is established, the effects of physical load and arm position on esophageal sphincter pressures remain unclear. This study investigated how postural demand and external load influence upper (UES) and lower (LES) esophageal sphincter pressures under varying stabilization conditions.

**Methods:**

Twenty-eight healthy adults underwent high-resolution manometry (HRM) to measure UES and LES pressures in standing, supine leg raise, and during standing while holding 3, 6, and 9 kg loads with arms alongside the body or raised to 45° shoulder flexion. Paired-samples *t*-tests and one-way ANOVA were applied, with effect sizes calculated.

**Results:**

LES pressure significantly increased in the supine leg raise and when loads were held with arms elevated at 45° shoulder flexion (*p* < 0.001), indicating enhanced activation of the esophagogastric high-pressure zone involving the crural diaphragm. No significant LES change occurred when weights were held parallel to the body. UES pressure significantly increased only during the supine leg raise (*p* < 0.001), whereas load magnitude had no effect in standing.

**Conclusion:**

Esophageal sphincter pressures are modulated by posture and load, supporting an integrated respiratory–postural–sphincteric function of the diaphragm. LES pressure rises with increasing postural demand, consistent with crural diaphragm recruitment, while UES responses appear position-dependent. These findings extend current understanding of the diaphragm’s coordinated role in esophagogastric junction and upper sphincter control.

## 1 Introduction

The diaphragm is considered a vital component of the postural control system and plays a key role in stabilizing the spinal column by creating intra-abdominal pressure, acting like a piston compressing the contents of the abdominal cavity (Sembera et al., 2022). As the diaphragm and abdominal muscles are antagonistic-synergistic structures, concentric contraction of the diaphragm induces eccentric activity of the abdominal musculature. This synergy facilitates the modulation of intra-abdominal pressure (IAP) to support spinal column stability (Hodges and Gandevia, 2000b). Beyond postural control and inspiratory function, the crural diaphragm serves as an extrinsic component of the lower esophageal sphincter (LES) complex, forming together with the intrinsic smooth muscle of the LES the esophagogastric high-pressure zone (HPZ) measurable by high-resolution manometry (Zheng et al., 2021).

In addition to its primary role in inspiration, the diaphragm is a crucial component of the core muscle complex (Wallden, 2017; Sannasi et al., 2023). Studies have shown that the diaphragm’s position and excursions change during postural loading and limb activities (Kolar et al., 2012; Vostatek et al., 2013; Sembera et al., 2022). In healthy individuals, caudal displacement and greater excursions of the diaphragm occur when lifting loads (Sembera et al., 2022). Diaphragm thickness and mobility are affected by body posture, with greater thickness observed in upright positions compared to supine (Hellyer et al., 2017). While Hodges observed that increased respiratory demand may reduce the diaphragm’s postural activity (Hodges et al., 2001), Sembera et al. (Sembera et al., 2022), in contrast, suggest that the diaphragm’s postural function remains independent of its respiratory role during load lifting. The diaphragm plays a crucial role in regulating IAP and spinal stability. Coactivation of the diaphragm and abdominal muscles causes sustained increases in IAP, while respiratory variations are controlled by opposing activity of these muscles (Hodges and Gandevia, 2000b). Sembera et al. (2022) state that during tidal breathing, the diaphragm does not significantly enhance spinal stiffness. However, when an individual is required to lift a heavy object while continuing to breathe, the diaphragm increases its excursion in a caudal direction. This is accompanied by increased tension in the abdominal wall, leading to elevated intra-abdominal pressure and providing stability to the spinal column. Numerous other studies confirm that IAP increases spinal stiffness and stability (Hemborg et al., 1985; Hodges et al., 2005; Arjmand and Shirazi-Adl, 2006; Novak et al., 2022); although a few older studies argue that IAP does not reduce spinal load (Nachemson et al., 1986; KUMAR, 1997).

While the dual respiratory-postural function of the diaphragm is frequently discussed in the scientific literature, with numerous studies available, albeit some presenting conflicting conclusions, the more complex respiratory-postural-sphincteric function of the diaphragm remains less thoroughly investigated. The crural diaphragm functions as an external component of the anti-reflux barrier. Its contractions enhance LES pressure during inspiration and in response to elevated abdominal pressure. However, these contractions are temporarily inhibited to allow the passage of gas or a bolus through the gastro-esophageal junction (van Herwaarden et al., 2004). In patients with gastroesophageal reflux disease (GERD), the sphincteric function of the diaphragm is compromised. Patients with GERD exhibit decreased LES pressure, reduced crural diaphragm tension, and greater LES-crural diaphragm separation compared to healthy individuals (Pandolfino et al., 2007).

In the context of GERD, effective concentric and eccentric diaphragm activity during inspiration and expiration is necessary to accommodate changes in intra-abdominal pressure. This requirement arises due to the natural pressure gradient between the thoracic and abdominal cavities, with the abdominal cavity exhibiting higher pressure comparatively (Tack and Pandolfino, 2018). This gradient becomes more prominent during inspiration, particularly loaded inspiration, when negative thoracic pressure is necessary for lung inflation. Simultaneously, caudal displacement of the diaphragm, coupled with eccentric contraction of the abdominal wall, increases and modulates intra-abdominal pressure for spinal stabilization. Without proper function of the LES and crural diaphragm, retrograde flow of gastric contents may occur (Tack and Pandolfino, 2018).

Dysfunction of the respiratory-postural function of the diaphragm may be one of the causes of low back pain (LBP) (Kolar et al., 2012; Vostatek et al., 2013), while impairment of its complex respiratory-postural-sphincteric function may contribute to GERD (Zdrhova et al., 2022). GERD and LBP frequently coexist, particularly in patients with chronic obstructive pulmonary disease (COPD) (Imagama et al., 2012; Bordoni et al., 2018). Additionally, studies have demonstrated a significant overlap between GERD and laryngopharyngeal reflux disease (LPRD). Patients with both conditions tend to experience the most severe reflux symptoms (Wang et al., 2023). Upper esophageal sphincter (UES) abnormalities are often observed in about one-third of LPRD patients, but these are not specific to LPRD (Benjamin et al., 2017) and the relationship between UES function and LPRD is not fully established (Lippincott and Velanovich, 2022).

This study investigated how different body positions and load-carrying conditions affect UES and LES pressures in relation to diaphragm activity, using high-resolution manometry (HRM). We hypothesize that arm positioning and load weight significantly influence sphincter function. By examining these interactions, the study addresses gaps in our understanding of the diaphragm’s integrated respiratory, postural, and sphincteric roles. The findings contribute to the broader field of gastroesophageal and postural physiology and offer insights relevant to the clinical management of GERD, LPRD, and related disorders. By combining HRM with biomechanical loading, this research enhances our knowledge of the neuromechanical regulation of esophageal sphincters and core stability, highlighting the interaction between neural control and musculoskeletal function under physiological demands. Given that the diaphragm is a skeletal muscle and thus under voluntary control, the insights gained may inform the development of targeted rehabilitation strategies aimed at improving the voluntary regulation of diaphragm and core muscle function. Such approaches could help restore or compensate for impairments in the diaphragm’s integrated respiratory, postural, and sphincteric functions, offering new therapeutic pathways for disorders associated with their dysfunction.

## 2 Materials and methods

### 2.1 Participants

Written informed consent was obtained from each participant. The study was approved by the Institutional Ethical Board of University Hospital Motol, Prague, Czech Republic (Ref. No. EK-1340.25/20). It was conducted in accordance with the Declaration of Helsinki and followed relevant CONSORT recommendations for transparent reporting of studies involving human participants.

Twenty-eight healthy participants (20 females and 8 males), aged 20–31 years, with weights ranging from 50 kg to 101 kg, heights from 159 cm to 194 cm, and body mass index (BMI) values between 17.5 and 29.0, were recruited for this study (Table 1). The study involved healthy individuals without any signs of GERD, digestive issues, back pain, or respiratory problems. The health status of these individuals was assessed using a questionnaire that confirmed the absence of symptoms or conditions related to these health concerns. The following questionnaires were used in their validated Czech versions: the Gastroesophageal Reflux Disease Questionnaire (GERDQ), the Oswestry Disability Index (ODI), and the COPD Assessment Test (CAT). The exclusion criteria included a history of chronic gastrointestinal and respiratory disorders, chronic pain or dysfunctions of the musculoskeletal system, previous surgeries involving these systems, acute pain, or any acute illness. All testing procedures were thoroughly explained to the participants with a detailed description of the assessments.

**TABLE 1:** Descriptive statistics of participants (Mean ± Standard Deviation).

Participants	Age (y)	Height (cm)	Weight (kg)	Body mass index
All (n = 28)	24.0 ± 2.5	171.1 ± 10.1	70.3 ± 11.0	23.2 ± 2.9
Females (n = 20)	23.5 ± 2.3	169.4 ± 6.7	66.3 ± 8.7	23.1 ± 2.8
Males (n = 8)	25.5 ± 2.4	186.0 ± 7.0	80.3 ± 10.0	23.3 ± 3.4

### 2.2 Assessments

Participants were instructed not to eat or drink for 2 h prior to the examination. High-resolution esophageal manometry (HRM) was performed using the Solar GI system (Medical Measurement Systems, Enschede, Netherlands) and a 36-channel solid-state catheter with circumferentially arranged pressure sensors at 1 cm intervals. The catheter was introduced transnasally with the participant initially in the seated position for anatomical identification. After localization of the upper and lower esophageal sphincters, the catheter was fixed, and the participant repositioned to the supine posture. A minimum 30-s baseline recording was obtained, followed by ten 5-mL water swallows at 30-s intervals. Pressure data were continuously recorded at a sampling rate of 10 Hz per channel, meaning each pressure sensor collected 10 measurements per second, allowing precise detection of rapid pressure changes during swallowing. Data were analyzed using MMS Solar GI software with standard filtering and automated averaging settings (Cohen and Shirin, 2023).

**FIGURE 1:**
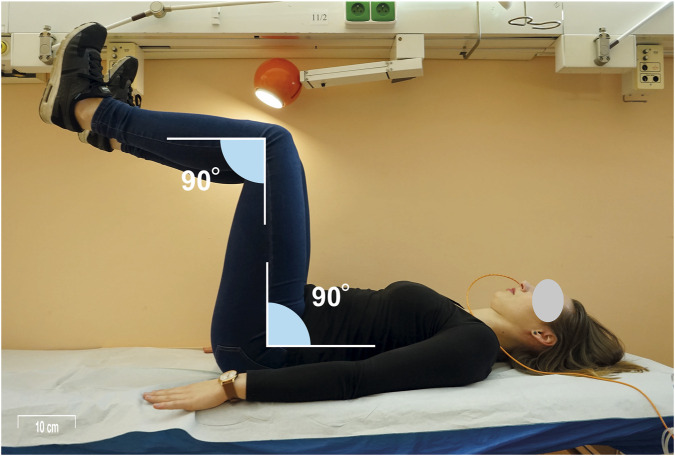
Measurement scenario 1: The participant actively maintained 90° flexion at the hips and knees in a supine position, following passive positioning by the clinician. UES and LES pressures were recorded in this posture. A HRM catheter was placed transnasally, as visible in the image.

**FIGURE 2:**
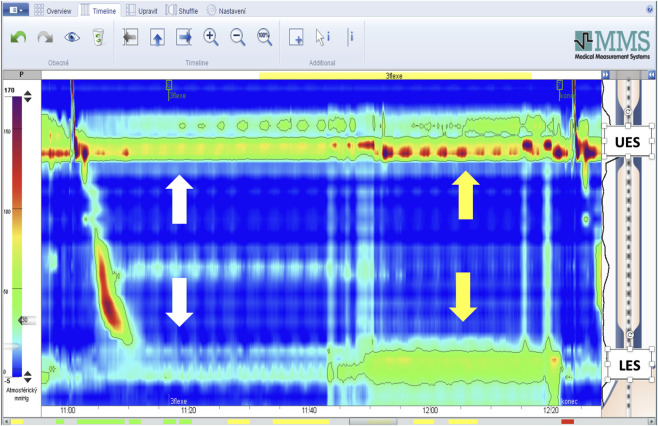
Representative HRM recording during supine leg raise. Upper arrows indicate the UES and lower arrows indicate the LES/HPZ. White arrows denote resting pressure, whereas yellow arrows denote increased sphincter pressure following elevation of the lower extremities (90° hip and knee flexion).

**FIGURE 3:**
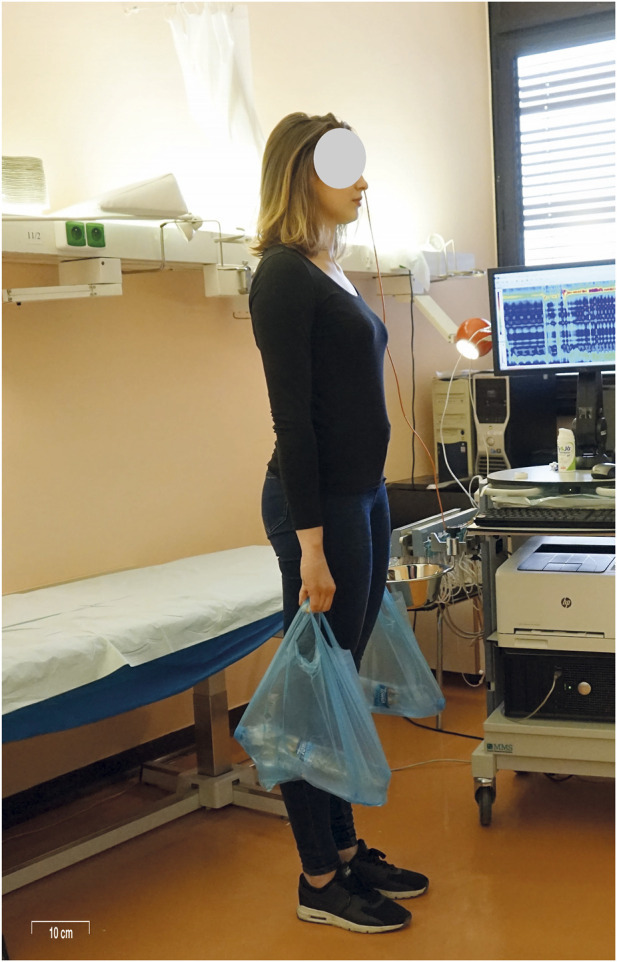
Measurement scenario 2: The participant held a 3–9 kg load with the arms extended naturally alongside the body, simulating a daily activity such as carrying grocery bags. This posture served as the control arm position for comparison with the elevated arm position shown in Figure 3. UES and LES pressures were recorded in this posture.

**FIGURE 4:**
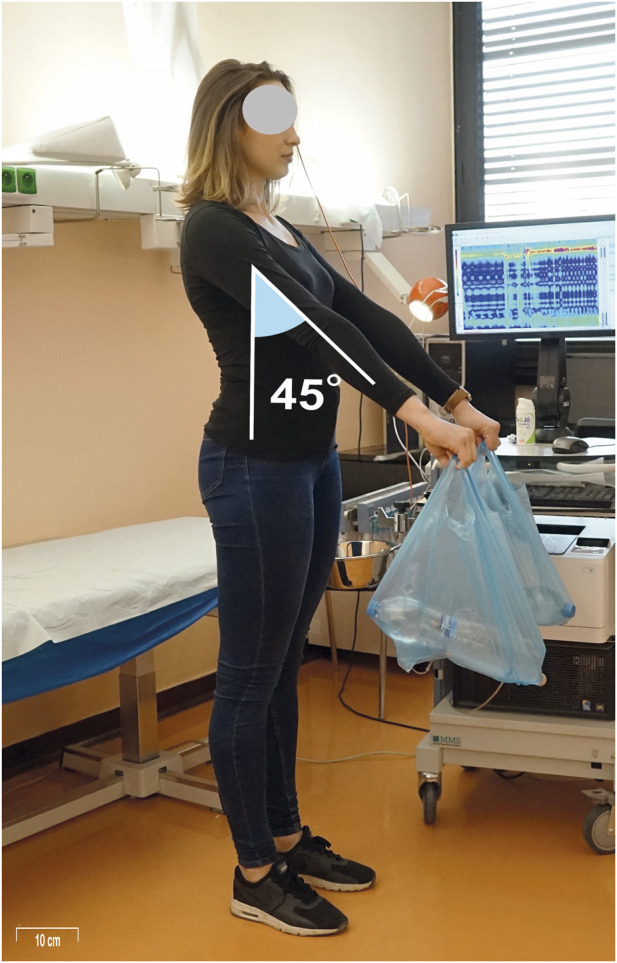
Measurement scenario 3: The participant held a 3–9 kg load with the arms extended in approximately 45° of shoulder flexion. Compared to carrying the load alongside the body, this posture shifts the load forward and increases the mechanical demand on trunk stabilization. It also reflects daily activities that involve briefly lifting or repositioning a load in front of the body, such as removing a bag from a car trunk or placing an object onto a table.

Following this, testing in various postural positions was initiated, with UES and LES/HPZ pressures recorded in each position as follows: (1) The clinician passively raised the participant’s lower extremities to 90 degrees of flexion at the hips and knees while in a supine position, after which the participant was instructed to actively maintain this posture for UES and LES pressure measurements (Figure 1) and the associated increase in UES and LES pressures is demonstrated in a representative HRM recording (Figure 2); (2)The participant adopted a standing position with the arms alongside the body and held a 3 kg load with the arm alongside the body (parallel to the torso (Figure 3); (3) The participant then held the same 3 kg load with the arm extended in 45° shoulder flexion. (Figure 4); (4) The participant held a 6 kg load with the arm alongside the body. (5) The participant then held the same 6 kg load with the arm extended in 45° shoulder flexion. (6) The participant held a 9 kg load with the arm alongside the body; (7) The participant then held the same 9 kg load with the arm extended in 45° shoulder flexion. UES and LES pressures were recorded after each change in load or arm position. The order of testing conditions was randomized across participants to minimize order effects. Blinding of the examiner was not feasible due to the nature of the experimental protocol, which required direct observation and participant instruction. Pressure data were analyzed using standardized HRM software algorithms to minimize subjective bias.

The most common load-bearing activity in today’s population is carrying groceries. Therefore, we chose to use 1.5 kg bottles placed in shopping bags as the load. The weight was always distributed evenly between both hands. In the case of a 3 kg load, a shopping bag containing a single 1.5 kg bottle of water was placed in each hand. For the 6 kg load, a shopping bag with two 1.5 kg bottles of water was placed in each hand. For the 9 kg load, a shopping bag with three 1.5 kg bottles of water was placed in each hand. Standing with the arm extended in approximately 45° shoulder flexion simulates a common daily activity, such as lifting a shopping bag and placing it onto a table in front. The participant maintained each position for 20 s.

All UES and LES pressure measurements were performed by the same experienced therapist under consistent conditions, using the same HRM apparatus and catheter type. Manometric data were analyzed using MMS Solar GI HRM software.

### 2.3 Statistical analysis

Descriptive statistics were calculated for all variables, and data are mean ± standard deviation, unless otherwise stated. The full de-identified dataset of UES and LES values are publicly available to download via Figshare at: https://figshare.com/articles/dataset/GERD_Master_Data_sav/29137562?file=54781622.

All variables were normally distributed, except a single outlier, as assessed by studentized residuals greater than ±3 standard deviations, and using ±1.96 as cutoffs for skewness and kurtosis (Kim, 2013) (respectively). The outlier was identified in only one variable, and was handled by winsorizing its value (transforming it to the next largest value, retaining the rank order) (Field, 2017) which improved normality of that variable. Paired-samples *t*-tests were used to compare esophageal standing resting pressure in both the UES and LES with the supine position and when holding different weights in the supine position, with effect sizes interpreted as *small* (<0.2), *medium* (0.5–0.8), or *large* (>0.8) as proposed by Cohen (Cohen, 1988). A one-way analysis of variance (ANOVA) was used to determine the effect of different weights held parallel and with arms outstretched at 45° of shoulder flexion on the UES and LES pressures. Power analysis, using G*Power 3.1, indicated an 80% chance of detecting a medium effect size of 0.5 in 27 subjects for the paired t-tests, and an 80% chance of detecting a medium effect of 0.06 (η^2^) in 28 subjects for the ANOVA, with statistical significance determined a priori at p < 0.05. Data analyses were conducted with the Statistical Package for the Social Sciences (SPSS version 29.0 for Mac; IMB Corp, Armonk, NY).

## 3 Results

**FIGURE 5:**
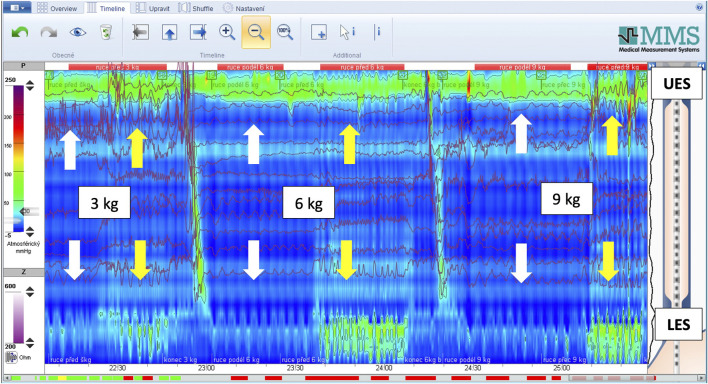
Representative HRM recording showing the effect of arm position and external load. Upper arrows indicate the UES and lower arrows the LES/HPZ. White arrows denote pressures recorded while the load was held with the arms alongside the body, whereas yellow arrows denote pressures recorded while the load was held in 45° shoulder flexion in front of the body. The magnitude of the load (3,6 and 9 kg) is indicated within the figure.

The paired-samples *t*-tests revealed several significant findings (Table 2). We observed a significant increase in both UES and LES values when participants were lying supine compared to the resting standing position (*p* < 0.001). For the LES, all three weights held with shoulders flexed to 45° in front of the body demonstrated significantly greater LES pressures than when the weights were held parallel with the body (*p* < 0.001–0.007), as illustrated by a representative high-resolution manometry tracing (Figure 5). For the UES, no differences were observed for any of the weights when comparing the 45° shoulder flexion position with arms held parallel (*p* = 0.238–0.398). The ANOVA analyses revealed no significant differences in either the UES or LES pressures across the different weights held (Table 3). BMI was significantly correlated with age (*r* = 0.49) and weight (*r* = 0.69), with no other significant findings to report.

**TABLE 2:** UES and LES pressure changes during postural tasks and varying loads (mean ± SD).

Measure	Resting	Supine position^a^	Mean difference	95% CI	Effect size (Cohen’s d)	*P* v*alue*
UES	65.25 (26.10)	118.32 (55.33)	−53.07	(-73.44, −32.70)	−1.01	<0.001*
LES	20.11 (9.02)	45.00 (20.05)	−24.89	(-32.30, −17.48)	−1.3	<0.001*
Holding 3 kg Parallel^b^ 45° shoulder flexion^c^						
UES	68.21 (25.64)	66.18 (19.74)	2.04	(-6.04, 10.11)	0.09	0.305
LES	22.14 (10.45)	27.18 (11.15)	−5.04	(-8.99, −1.08)	−0.49	0.007
Holding 6 kg						
UES	69.18 (21.77)	68.43 (21.53)	0.75	(-5.17, 6.67)	0.05	0.398
LES	21.46 (9.60)	28.07 (11.00)	−6.61	(-9.03, −4.19)	−1.06	<0.001**
Holding 9 kg						
UES	76.18 (25.13)	73.71 (19.77)	2.46	(-4.51, 9.45)	0.14	0.238
LES	24.36 (10.22)	33.18 (13.92)	−8.82	(-12.65, −4.99)	−0.89	<0.001**

^a^ Subject actively held lower extremities to 90° of hip and knee flexion.

^b^ Subject holds load with arms parallel to their torso.

^c^ Subject holds the same weight with the arms raised into 45° of shoulder flexion.

Note: UES: upper esophageal sphincter; LES: lower esophageal sphincter.

Effect size = calculated Cohen’s d.

*Statistically significant difference observed (*P* < 0.05).

**Statistically significant difference observed (Bonferroni Correction *P* < 0.016).

**TABLE 3:** ANOVA results for UES and LES by weight and arm position in standing posture. Total n = 28.

	Measure	3 kg	6 kg	9 kg	df	F	*P value*	*η2*
Parallel^a^	UES	68.21 (25.64)	69.18 (21.77)	76.18 (25.13)	2	0.9	0.41	0.02
	LES	22.14 (10.44)	21.46 (9.60)	24.36 (10.22)	2	0.63	0.54	0.02
45° shoulder flexion^b^	UES	66.18 (19.74)	68.42 (21.53)	73.71 (19.77)	2	1.01	0.37	0.02
	LES	27.18 (11.15)	28.07 (10.96)	33.17 (13.92)	2	2.01	0.14	0.05

^a^ Subject was standing holding weight parallel with their torso.

^b^ Subject was standing holding weight with arms outstretched to 45° Shoulder Flexion.

## 4 Discussion

This study demonstrated that esophageal sphincter pressures are modulated by posture and external loading in healthy adults. Using HRM, we found that LES pressure significantly increased during the supine leg raise and when loads were held with the arms elevated to 45° shoulder flexion, both conditions requiring enhanced postural stabilization. In contrast, no significant LES pressure change occurred when loads were held alongside the body. UES pressure increased only during the supine leg raise, suggesting a position-related protective response. These findings indicate that activation of the crural diaphragm contributes to postural modulation of LES pressure, supporting its integrated respiratory-postural-sphincteric role.

While HRM allows real-time monitoring, it is not always possible to accurately distinguish between pressure generated by the crural diaphragm and the circular smooth muscle of the LES, due to anatomical overlap within the sphincter complex. The maneuvers in our study involved lifting the lower limbs with hips and knees flexed to 90° and feet elevated off the supporting surface, and holding various weights either with arms alongside the body (simulating carrying a shopping bag) or with arms outstretched at 45° shoulder flexion. In the supine position, our results indicated that lower limb elevation causes a significant increase in LES pressure (Figure 2), aligning with findings from Bitnar et al. (Bitnar et al., 2015). The increase in LES pressure is largely attributable to the crural diaphragm activation (Mittal, 1990). These findings confirm the diaphragm’s postural function, consistent with imaging-based evidence (Kolar et al., 2010; 2012; Vostatek et al., 2013).

The diaphragm also responded when a load was held in front of the body (in front of the center of gravity), whereas when the arms were held alongside the body (closer to the center of gravity), LES pressure did not increase, indicating that the diaphragm was not recruited for axial stabilization (Figure 5). This likely reflects the higher postural demand when the load is positioned anteriorly, requiring the diaphragm to generate intra-abdominal pressure (IAP) in coordination with the abdominal wall to stabilize the thoracolumbar region and maintain trunk alignment. This supports the postural function of the diaphragm, suggesting that its involvement is recruited only under conditions of increased postural demand, particularly when axial segments are threatened by destabilization (e.g., leverage stress or fall risk). Under such conditions, the diaphragm contributes to postural stabilization, which is visible as increased LES pressure on HRM. A trend was also observed: the heavier the load, the greater the pressure and thus crural diaphragm activation at the LES. However, the pressure differences between holding 3, 6, and 9 kg loads were not statistically significant, though the trend was consistent. These findings indicate that the diaphragm is integrated into central postural-motor control strategies, functioning as a postural muscle that contributes to anticipatory and compensatory stabilization mechanisms.

Anticipatory and compensatory stabilization mechanisms rely heavily on the diaphragm’s contribution to spinal control. Given the crural diaphragm’s insertion on the lumbar spine (Khadanovich et al., 2025), stabilizing this region is essential to provide a stable anchoring point and enhance the diaphragm’s force-generating capacity. Caudal displacement of the diaphragm during tidal and loaded breathing has been demonstrated using MRI and ultrasound imaging (Kolar et al., 2010; Sembera et al., 2022). In one study, resistance was applied externally during isometric straight leg raises (Kolar et al., 2010), which challenge the system less than hip flexion with extended knees due to the shorter lever arm. Loaded breathing resulted in significantly greater caudal displacement during inspiration and a lower expiratory diaphragm position compared to unloaded conditions, suggesting residual tonic diaphragmatic activity for intra-abdominal pressure regulation and spinal stabilization. The study also demonstrated that the diaphragm does not act as a uniform structure during loaded breathing, with the greatest displacement occurring in its middle and posterior regions, particularly the crural portion (Kolar et al., 2010). Although several studies have addressed the diaphragm’s role in postural control and displacement (Kolar et al., 2010; 2012; Sembera et al., 2022), evidence regarding the specific pressure contributions of the crural diaphragm during postural challenges remains limited. The rapid, task-dependent LES pressure responses observed in our study are consistent with anticipatory postural activation of the diaphragm, as described in previous work by Hodges (Hodges and Gandevia, 2000a) and Kolar (Kolar et al., 2010).

In standing, LES pressure significantly increased when a load was held with arms in 45° shoulder flexion and extended elbows, likely reflecting concentric diaphragm activation to raise intra-abdominal pressure in response to greater postural demand, particularly as the load was positioned away from the body’s center of mass. In contrast, no significant difference in LES pressure was observed when 3, 6, or 9 kg were held alongside the body, indicating no additional postural stabilization demand. Although we hypothesized that diaphragmatic engagement and thus LES pressure would proportionally increase with load, no significant differences were found among the 3, 6, or 9 kg loads held at shoulder height. This absence of a linear dose–response pattern likely reflects a physiological ceiling effect rather than a statistical limitation: in healthy individuals, LES pressure appears to rise once a stabilization threshold is reached, beyond which additional loading does not elicit further increases. Inter-individual variability in stabilization capacity may also contribute, as our sample included both males and females of varying ages and fitness levels, with a fixed load applied across participants. The consistent manometric responses, together with adequate statistical power to detect medium effects, support the interpretation that the observed plateau represents a true physiological phenomenon. Future studies should clarify the relationship between load magnitude and diaphragmatic recruitment in the HPZ, particularly in clinical populations with impaired LES function. Moreover, a sedentary individual may require greater muscular effort than an active one, and sex-related differences in stabilization strategies may exist given that males generally have greater muscle mass (Nuzzo, 2024).

Future studies might benefit from using load relative to body weight, as done by Sembera et al. (2022), who used approximately 20% of body mass. However, their protocol involved bent elbows, which is biomechanically less demanding than the 45° shoulder flexion used here. Replicating our posture with 20% body weight may therefore be too challenging for some participants, particularly females with higher BMI.

Participants likely employed different postural stabilization strategies. According to Kolar et al. (2012), superficial back extensors can substitute for diaphragmatic stabilization, but this results in hyperlordosis, anterior pelvic tilt, and reduced spinal stiffness (Hodges et al., 2005). Such compensatory alignment increases facet and disc loading (Lv et al., 2016) and alters breathing patterns toward accessory muscle dominance (Zdrhova et al., 2022), potentially inhibiting diaphragmatic sphincter function. The vertical alignment of the diaphragm over the pelvic floor is essential for optimal contraction (Zdrhova et al., 2022); deviations from this alignment could explain the absence of LES pressure differences between 3, 6, and 9 kg loads. Because participants were not instructed to use a specific stabilization strategy, postural variability may have masked subtle load-dependent changes. Future studies should therefore combine HRM with postural tasks of varying load and duration to assess the reproducibility of HPZ pressure responses. Sample size calculations should be based on pilot-derived effect sizes, and imaging modalities such as dynamic upright MRI or ultrasonography could further elucidate diaphragm and esophagogastric junction behavior during loading.

Regarding upper esophageal sphincter (UES) responses, the literature is limited. In our experiment, UES pressure significantly increased during supine leg raise. The UES prevents aerophagia and protects the airway from aspiration during reflux events. Therefore, this pressure increase may represent a protective response related to body position. Supporting this, no significant UES pressure change occurred when participants held a load alongside the body or with arms at 45° shoulder flexion. Unlike the supine position, where proportional esophageal sphincter activation occurred during leg raise, the standing posture likely places less demand on UES activity, potentially due to gravitational assistance in bolus transfer. In this position, spinal stabilization is managed by axial and abdominal muscles, including the diaphragm, without significant UES involvement, given the orientation of the cricopharyngeus and thyropharyngeus muscles forming the UES (Ramaswamy et al., 2022).

Our results are consistent with findings from Bitnar et al. (2015), who also observed increased UES pressure during supine leg raise. Interestingly, participants with higher resting UES pressure demonstrated greater increases during leg raise compared to those with lower resting pressure. This may be due to irradiation of muscle activity from neck extensors and suprahyoid/infrahyoid muscles. According to proprioceptive neuromuscular facilitation (PNF) principles, large muscle group activity can enhance smaller muscle contractions, supporting therapeutic strategies for strengthening (Gontijo et al., 2012). In line with Dynamic Neuromuscular Stabilization (DNS), individuals with insufficient diaphragm-abdominal coordination may compensate via substitute stabilization strategies, such as increased upper trapezius activity, altering spinal curvature, a phenomenon seen in the “open scissors” syndrome (Kobesova et al., 2014). Similar patterns may occur in supine leg raise when neck flexor-diaphragm coordination is impaired, leading to excessive activation of superficial neck extensors. This may explain the higher UES tension in participants with elevated baseline pressures, as noted in study by Bitnar et al. (2015). Supporting this, Bitnar’s team also demonstrated that manual cervical traction significantly reduced UES pressure in supine participants (Bitnar et al., 2021). Traction may aid in maintaining neutral cervical alignment, providing a stable fixing point for surrounding muscles and promoting relaxation. Therapeutically, cervical traction alleviates radicular symptoms by enlarging neural spaces and reducing compression (Vanti et al., 2021). Additionally, muscle stretching through traction may modulate gamma loop activity, decreasing muscle spindle sensitivity and thus reducing tone (Avela et al., 1999). Therefore, alterations in UES pressure may be partially influenced by the tension of surrounding musculature.

Clinically, these findings are important because they open a potential pathway for influencing LES function through targeted physical exercise. We showed that the diaphragm responds not only to lower limb elevation but also to upper limb activation, which is a novel observation with direct relevance for physiotherapeutic practice. This knowledge can be applied to exercise strategies that combine limb loading with deep breathing to enhance diaphragmatic contribution to LES pressure, in line with emerging trends in physiotherapy for reflux management (Qiu et al., 2020). To date, no studies have examined the effect of diaphragmatic activation on LES function using loaded exercise in the upright position. Our results indicate that this is feasible and suggest that improving the conditioning of the diaphragm and abdominal muscles may provide a means to modulate LES pressure. Previous studies have shown that a substantial proportion of GERD patients experience reflux predominantly in the upright position during daily activities, referred to as upright reflux (Hoppo et al., 2012). Given this, upright stimulation of LES pressure should be considered alongside supine interventions. Establishing these physiological response patterns in a healthy young population provides a reference for identifying deviations in patients with GERD and for developing non-pharmacological rehabilitation approaches aimed at improving the function of the esophagogastric junction. The UES response observed during supine leg raise may also be relevant for understanding cervical pharyngeal coactivation and could inform rehabilitation strategies in patients with impaired postural respiratory coordination, for example, through targeted training of cervical–diaphragmatic interaction or postural-respiratory exercises.

Although the study was conducted in healthy young adults, the observed respiratory, postural, and sphincteric interactions may provide a physiological reference for understanding dysfunction in clinical populations, such as individuals with GERD, LPRD, or postural disorders. Age-related changes in tissue properties, respiratory mechanics, and postural control may further influence these relationships. Therefore, extrapolation to older or clinical populations should be made with caution, and future studies are needed to confirm whether these mechanisms are preserved or altered in these groups.

Several methodological and design-related limitations should be considered when interpreting the results of this study. First, the small sample size (n = 28), gender imbalance, and narrow age range limit generalizability. Although the repeated-measures design increased statistical power and recruitment of healthy young adults reduced confounding from age-related comorbidities, applicability to clinical populations (e.g., GERD, LPRD, postural disorders) remains limited. Second, the use of uniform absolute loads (3, 6, 9 kg) simplified the protocol and reflected daily activities such as carrying groceries but introduced variability in postural demand. Future studies should therefore include broader age groups, balanced gender representation, relative loading strategies (e.g., % body weight or strength), and clinical populations. Third, while HRM enables pressure monitoring in the esophageal sphincters, it cannot distinguish between crural diaphragm activity and circular smooth muscle pressure. The absence of concurrent imaging (e.g., ultrasound or MRI) limits our ability to confirm diaphragm displacement or recruitment patterns during testing. It may be more precise to describe our findings as changes within the HPZ of the esophagogastric junction, which represents a functional unit composed of both the intrinsic smooth muscle of the LES and the extrinsic crural diaphragm (Kahrilas and Nguyen, 2025). However, the temporal characteristics of the responses provide indirect evidence supporting primarily crural diaphragm activation. Specifically, the very rapid phasic increases and decreases in LES/HPZ pressure observed in our study are more consistent with skeletal muscle activity of the crural diaphragm than with the tonic contractile behavior of smooth muscle. This interpretation aligns with prior work by Kolar et al. (2010) and Hodges and Gandevia (2000a), who demonstrated diaphragm recruitment during postural tasks using imaging (MRI) and EMG methods. Nonetheless, we acknowledge that this remains inferential, and concurrent imaging or direct measures of diaphragmatic motion would strengthen future investigations. Fourth, participants were not instructed to use a specific postural stabilization strategy, which may have introduced variability in motor patterns. Finally, the tasks were of short duration (20 s) and do not reflect sustained or fatiguing conditions, which could differently affect muscle recruitment and pressure regulation. The 20-s interval was chosen because most participants were unable to maintain the postures longer without pain or compensatory changes (e.g., increased lumbar lordosis, dropping of the arms). Future studies should therefore incorporate longer-duration or fatigue protocols to better understand sustained diaphragmatic responses to postural challenges.

## 5 Conclusion

This study demonstrates that esophageal sphincter pressures, particularly within the lower esophageal sphincter (LES) and esophagogastric high-pressure zone (HPZ), vary with body position and external loading in healthy adults. LES pressure increased when tasks required greater postural stabilization, while upper esophageal sphincter (UES) pressure rose primarily during the supine leg raise. These patterns indicate coordinated modulation of sphincter tone associated with postural demand, likely reflecting recruitment of the crural diaphragm and synergistic muscle groups involved in trunk stabilization.

The findings advance the concept of an integrated respiratory–postural–sphincteric function of the diaphragm and provide quantitative evidence of neuromechanical coupling within the esophagogastric junction and pharyngoesophageal segment. Future work integrating high-resolution manometry with imaging or electromyographic techniques could further delineate the relative contributions of diaphragmatic and sphincteric components and clarify their physiological and clinical relevance.

## Data Availability

The full de-identified dataset of UES and LES values are publicly available to download via Figshare at: https://figshare.com/articles/dataset/GERD_Master_Data_sav/29137562?file=54781622.
